# Theoretical integration of user satisfaction and technology acceptance of the nursing process information system

**DOI:** 10.1371/journal.pone.0217622

**Published:** 2019-06-04

**Authors:** Kuei-Fang Ho, Cheng-Hsun Ho, Min-Huey Chung

**Affiliations:** 1 School of Nursing, College of Nursing, Taipei Medical University, Taipei, Taiwan; 2 Graduate Institute of Information Management, National Taipei University, New Taipei City, Taiwan; 3 Department of Nursing, Shuang Ho Hospital, Taipei Medical University, New Taipei City, Taiwan; Robert Gordon University, UNITED KINGDOM

## Abstract

**Background:**

The nursing process system (NPS) is used to establish the nursing process involving assessment, diagnosis, planning, intervention, and evaluation in solving the health problems of patients.

**Objectives:**

The factors influencing the use of the NPS by nurses were analyzed based on user satisfaction and technology acceptance within the 3Q (service quality, information quality, and system quality) model.

**Methods:**

In this cross-sectional quantitative study, the valid responses of 222 nurses to a questionnaire were obtained; these nurses worked at eight hospitals affiliated with public organizations in Taiwan. Structural equation modeling was used to analyze information quality, system quality, service quality, user satisfaction, perceived usefulness, perceived ease of use, perceived enjoyment, behavioral attitude, and intention after the nurses had used the NPS system for more than 1 month.

**Results:**

Information quality, service quality, and system quality influenced user satisfaction. User satisfaction affected perceived usefulness, perceived ease of use, and perceived enjoyment and had the highest explanatory power (R^2^ = 0.75). Furthermore, perceived usefulness, perceived ease of use, and perceived enjoyment influenced behavioral attitude and intention to use the system. The proposed model explained 53% of the variance in the intention to use the NPS.

**Conclusions:**

The relationships between the variables of the 3Q model were successfully used to examine the intention of nurses toward using the NPS. Using the findings of this study, designers and programmers can comprehensively understand the perceptions of nurses and further improve the performance of the NPS.

## 1 Introduction

Nurses manage electronic health and clinical care records in routine practice; their usage of electronic health records has correspondingly increased to up to 80% [1]. The nursing information system (NIS) is crucial to delivering eHealth services [2], and this system comprises an integrated module of electronic health records [3]. The NIS can provide technological assistance for the management of all aspects of a task and can improve workflow efficiency [3–5]. The nursing process system (NPS) is a part of the NIS and is widely used by nurses to improve the quality of care. The NPS is different from decision support systems, which help nurses make decisions. The NPS involves assessment, diagnosis, planning, intervention, and continual evaluation of the effectiveness of the patient care plan by using information technology. The nursing process is the core of practice through which nurses from various domains deliver holistic and patient-focused care [6].

The challenges of using the NIS include operational failures and mismatches between the process flow of the system and the workflow of nurses [3, 7]. NPS programmers, who do not have medical experience, rely on senior nurses’ experience and knowledge to analyze the practical nursing workflow and nursing documents when completing interface design and programming. Therefore, programmers who design, troubleshoot, and maintain the NPS must consider nurses’ opinions for the successful implementation of the NPS. Users have positive perceptions of using these systems to enhance work performance [8]. Understanding the factors that influence the intention of professionals to use the NPS is crucial [9].

The Wixom and Todd (WT) model involves a combination of user satisfaction (US) and technology acceptance, which are the two primary research streams for investigating the perception of information system success [8]. Within the WT model, US comprises object-based beliefs (information quality [IQ] and system quality [SysQ]) and object-based attitudes (information satisfaction and system satisfaction). Technology acceptance of WT model comprises behavioral beliefs (perceived usefulness [PU] and perceived ease of use [PEOU]), behavioral attitude (BA), and intention. IQ is an indicator of a user’s perception of the quality of the system’s conveyance of semantic meaning or communication of knowledge. For instance, the degree to which the information obtained from system is error-free is defined as the accuracy in IQ. SysQ is a user’s evaluation of the information system’s capabilities, the usability of the system, and the way of delivering information. For example, whether the system is readily accessible during a task is a part of SysQ. Information satisfaction and system satisfaction represent a user’s attitude toward the information system, and this attitude is primarily measured using IQ and SysQ. PU represents the behavioral belief that the information provided by an information system enhances work performance. Whether a system is judged to be easy to use is named PEOU. The BA construct represents a user’s point of view toward the usage of the technology. In summary, object-based attitudes are external variables that influence behavioral beliefs, and behavioral beliefs have been found to influence BA and intention [8].

Xu et al. [10] added SQ, service satisfaction, and perceived enjoyment (PE) to the WT model and explored the relationships between SQ, IQ, and SysQ to construct a 3Q model. They asserted that SQ depends on users’ overall evaluation of an information system and their opinions regarding the provided service. For example, the personal attention provided by a system in response to users’ concerns and specific needs during tasks is defined as empathy and responsiveness in SQ. Service satisfaction is an object-based attitude and is a cognitive and emotional reaction to SQ. PE indicates whether a user finds the system enjoyable, entertaining, and interesting to use [11]. Moreover, the 3Q model emphasizes the impact of service satisfaction on PU and PE in addition to how PE influences PEOU and BA.

**Fig 1:**
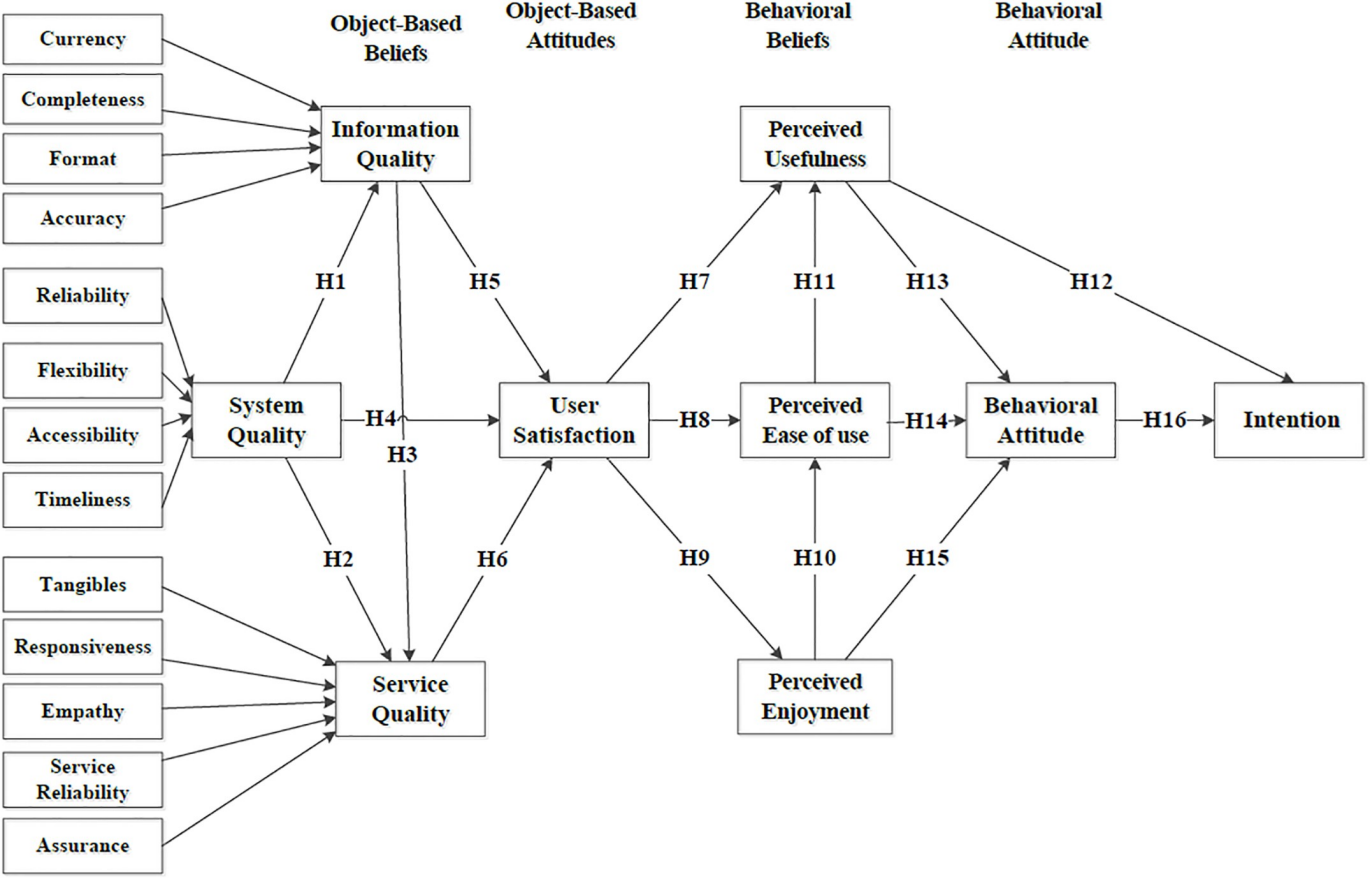
The 3Q model adopted in this study.

Extensive research has been conducted regarding the acceptance of and intention to use medical information technology [12–15]. Previous studies focused on certain characteristics of the behavioral intention regarding the use and acceptance of the information system by healthcare professionals [16, 17]. Some studies have investigated the influence of SQ, IQ, and SysQ with technology acceptance on US [9], the relationship between perceived quality and intrinsic individual perceptions [16], and the individual effects of IQ and SysQ on US [17]. However, behavioral beliefs were not considered, the comprehensive effects of 3Q (i.e., IQ, SysQ, and SQ) was not determined, and the effects of 3Q model for using the NPS were not explored. In accordance with the North American Nursing Diagnosis Association standard, the NPS investigated in this study was developed by a public organization and was validated in a previous study [4]. In the present study, we used the 3Q model as the framework and employed structure equation modeling (SEM) to examine the contextual factors underlying the intention of nurses to use the NPS. Associations between object-based beliefs (SQ, IQ, and SysQ), object-based attitude (US), behavioral beliefs (PE, PEOU, and PU), and BA and the effects of these variables on the intention to use the NPS were also examined. The assumption model adopted in this study is displayed in Fig 1.

## 2 Materials and methods

### 2.1 Theoretical framework

Based on US within the 3Q model, this study hypothesized the relationships between SysQ and IQ (H1), SysQ and SQ (H2), and IQ and SQ (H3). The success of an information system is determined by the users’ satisfaction with the overall quality of the system [18]. Delone and McLean [19] proposed that IQ, SysQ, and SQ affect US. Therefore, we combined service satisfaction, information satisfaction, and system satisfaction to determine US with the NPS. We hypothesized the research framework for SysQ (H4), IQ (H5), and SQ (H6) to determine US with the NPS. On the basis of the object-based attitude affected by behavioral beliefs, we hypothesized the relationships between US and PU (H7), PEOU (H8), and PE (H9). According to technology acceptance in the 3Q model, we hypothesized the relationships of PE with PEOU (H10) and PEOU with PU (H11). In addition, on the basis of the influence of behavioral beliefs on BA and intention, we hypothesized the relationships of BA with PU (H13), PEOU (H14), PE (H15), and intention (H16).

### 2.2 Sample

This study recruited nurses from eight hospitals affiliated with public organizations in Taiwan. The NPS used by the nurses had not been changed in the previous 6 months. The inclusion criteria for the participants were as follows: (1) older than 20 years, (2) with a nursing license, (3) consenting to participate, and (4) having used the NPS for more than 1 month. According to Cohen [20], when considering three of the maximum number of predictors, a minimum sample size of 76 is required to achieve a statistical power of 0.8 and a medium effect size of 0.15 with *p* < 0.05. The minimum sample size was 200 for partial-least-squares (PLS)-SEM [21, 22], and an attrition rate of 25% was expected; thus, we recruited 250 nurses. After excluding participants with missing data (n = 28), this study included the data of 222 nurses obtained from questionnaire surveys.

### 2.3 Design and measures

In this cross-sectional study, a questionnaire (S1 and S2 Files) was used to understand the nurses’ experiences and intentions regarding the NPS. The research questionnaire was based on the 3Q model proposed by Xu et al. [10]. The 3Q model is an extension of the theoretical integration of US and technology acceptance [8].

After obtaining approval from the original author [10], we employed a structured 81-item questionnaire assessing the perspective of nurses. The questionnaire evaluated (1) object-based beliefs of SQ, such as the empathy, service reliability, tangibility, assurance, and responsiveness of the service delivered by the NPS; (2) object-based beliefs of SysQ, including the reliability, accessibility, timeliness, and flexibility of the NPS; (3) object-based beliefs of IQ, such as the currency, completeness, format, and accuracy of the NPS; (4) object-based attitudes of US; (5) behavioral beliefs, such as PU, PEOU, and PE; and (6) BA and intention. All items of the questionnaire based on the 3Q model were measured using a Likert scale ranging from −5 (completely disagree) to 5 (completely agree), with 0 as the neutral score. The internal consistency of the variables was 0.71–0.97, and composite reliability (CR) was 0.84–0.98 [10]. Discriminant validity also satisfied, as the square root of the average variance extracted (AVE) exceeded inter-construct correlations [10].

### 2.4 Ethics and data collection

The self-reported questionnaire was collected from NPS users. By investigating the nurses’ experiences of using the information system, we explored the relationships between the variables in the research framework. We considered that the NPS was utilized by the staff for assessing patient signs and symptoms. We also explored the etiology of nursing diagnoses and determined the nursing outcomes and interventions for analyzing the perceptions of the nurses. This research plan was approved by the Medical Ethics Committee of Tri-Service General Hospital (TSGHIRB No B-104-13). We explained the procedure and theme of the study to the participants. After the participants provided written consent, we collected the questionnaires and entered the data into a computer. We provided gifts to the nurses who participated in this study.

### 2.5 Data analysis

The sociodemographic variables of the nurses and usage characteristics of the information system were analyzed using SPSS version 20.0 (Chicago, IL, USA) for Windows. PLS-SEM software was used to assess the statistical data and test the hypotheses. We used SmartPLS version 3.0 (University of Hamburg, Germany) to evaluate the causal model and perform confirmatory factor analysis.

The validity and reliability of the measurement model were assessed by determining internal consistency reliability, indicator reliability, convergent validity, and discriminant validity [23]. We also employed a Cronbach’s α higher than the recommended value of 0.7 to indicate internal consistency [24]. Hair, Ringle [23] suggested the following guidelines for model evaluation using SmartPLS: (a) internal consistency reliability: CR > 0.7; (b) indicator reliability: factor loading > 0.7; (c) convergent validity: AVE > 0.50; and (d) discriminant validity: the square root of the AVE for each construct should be higher than all of their cross loadings. To avoid multicollinearity, we applied the criterion that the coefficients of correlation between two variables must be <0.85 [25].

SEM is the appropriate statistical methodology for analyzing multivariate models [10]. We used SEM to estimate the variance of perceived IQ, SysQ, and SQ of the NPS based on the experimental design. The nonparametric bootstrapping procedure was employed to test the hypotheses and analyze path coefficients. Path coefficients were obtained by bootstrapping 222 cases and 5,000 samples. The structural model comprised path coefficients, R^2^ values of the dependent variables, and *p* values. The significance of path coefficients for *p* values was considered. If the significance indicator value was less than 0.05, the variable was concluded to have considerable influence.

Henseler, Dijkstra [26] suggested the use of the standardized root-mean-square residual (SRMR) for assessing the goodness of fit of the structural model. In this study, the SRMR was thus used to determine the goodness of fit for the PLS-SEM by using the average magnitude of the observed correlation and model-implied correlation matrix. We assessed the goodness of fit of the PLS path models according to the suggestions of Hu and Bentler [27]. The SRMR was lower than 0.08, which indicated a good model fit [26]. Low, moderate, and high levels of explanatory power are represented by R^2^ values of 0.25, 0.50, and 0.75, respectively [23].

## 3 Results

### 3.1 Participant characteristics

Most of the participating nurses were women (95.5%). More than half (54.05%) had a bachelor’s degree in nursing, 32.43% (n = 72) had less than 6 years of clinical experience, 85.59% had less than 6 years of hospital information system experience, and 63.51% did not experience pressure when using a computer (Table 1).

**Table 1:** Demographics of the participants (n = 222).

Variable	Sample Size	%
**Gender**		
Male	10	4.50
Female	212	95.50
**Education level**		
Senior vocational school	4	1.80
Associate degree	88	39.64
Bachelor’s degree	120	54.05
Master’s degree	10	4.50
**Position**		
Staff	215	96.85
Supervisor	7	3.15
**Total experience (y)**		
0–5	72	32.43
6–10	61	27.48
11–15	44	19.82
16–20	28	12.61
>20	17	7.66
**Experience of using Hospital Information System (y)**		
0–5	190	85.59
6–10	21	9.46
>10	11	4.95
**Experiencing pressure when using a computer**		
Yes	81	36.49
No	141	63.51

### 3.2 Measurement model

Table 2 lists the Cronbach’s α, factor loading, CR, and AVE values. In this study, the Cronbach’s α was between 0.71 and 0.95 for the variables. The CR ranged from 0.87 to 0.97. These values were higher than the established acceptance levels, which indicated high internal consistency. The factor loadings of all the items were greater than 0.7. The AVEs of the study variables were between 0.72 and 0.91. The factor loadings implied satisfactory indicator reliability, and the AVEs of all the constructs indicated satisfactory convergent validity. Table 3 details the correlation coefficients and AVE values. In this study, the square roots of the AVE for each variable were higher than the corresponding correlations, and all bivariate correlations had coefficients less than 0.85. Thus, the discriminant validity criteria were satisfied.

**Table 2:** Factor loadings, CR, and AVE for the study variables.

Construct	Factor Loadings	CR	AVE	Cronbach’s α	R^2^
Currency	0.81–0.95	0.91	0.77	0.85	
Completeness	0.85–0.94	0.92	0.79	0.87	
Format	0.92–0.94	0.93	0.86	0.84	
Accuracy	0.91–0.92	0.91	0.84	0.81	
Information Quality	0.92–0.95	0.95	0.87	0.92	0.74
Reliability	0.80–0.90	0.88	0.72	0.80	
Accessibility	0.89–0.93	0.94	0.84	0.90	
Flexibility	0.93–0.95	0.91	0.83	0.80	
Timeliness	0.85–0.89	0.91	0.76	0.84	
System Quality	0.95–0.96	0.95	0.91	0.90	0.56
Empathy	0.91–0.92	0.91	0.84	0.81	
Service Reliability	0.84–0.95	0.91	0.78	0.86	
Tangible	0.93–0.94	0.95	0.87	0.93	
Assurance	0.91–0.92	0.94	0.84	0.90	
Responsiveness	0.93–0.94	0.93	0.88	0.86	
Service Quality	0.84–0.91	0.87	0.77	0.71	0.75
User Satisfaction	0.80–0.89	0.89	0.72	0.81	0.75
Perceived Enjoyment	0.94–0.95	0.96	0.89	0.94	0.42
Perceived Ease Of Use	0.78–0.92	0.91	0.73	0.87	0.61
Perceived Usefulness	0.91–0.94	0.96	0.86	0.94	0.59
Behavioral Attitude	0.92–0.95	0.95	0.87	0.93	0.66
Intention	0.94–0.96	0.97	0.90	0.95	0.53

CR = composite reliability; AVE = average variance extracted; CUR = Currency; COM = Completeness; FOR = Format; ACU = Accuracy; IQ = Information Quality; REL = Reliability; ACE = Accessibility; FLE = Flexibility; TIM = Timeliness; SysQ = System Quality; EMP = Empathy; SER = Service Reliability; TAN = Tangible; ASS = Assurance; RES = Responsiveness; SQ = Service Quality; US = User Satisfaction; PEOU = Perceived Ease of Use; PU = Perceived Usefulness; PE = Perceived Enjoyment; BA = Behavioral Attitude

**Table 3:** Correlation coefficients and AVE for the study variables.

**Variable**	**1**	**2**	**3**	**4**	**5**	**6**	**7**	**8**	**9**	**10**	**11**	**12**	**13**	**14**	**15**	**16**	**17**	**18**	**19**	**20**	**21**	**22**
1. CUR	**0.88**																					
2. COM	0.62	**0.89**																				
3. FOR	0.64	0.80	**0.93**																			
4. ACU	0.62	0.72	0.77	**0.92**																		
5. IQ	0.65	0.79	0.76	0.76	**0.93**																	
6. REL	0.65	0.70	0.65	0.60	0.67	**0.85**																
7. ACE	0.76	0.69	0.67	0.63	0.71	0.70	**0.91**															
8. FLE	0.68	0.65	0.62	0.63	0.72	0.64	0.78	**0.91**														
9. TIM	0.77	0.63	0.62	0.62	0.62	0.60	0.75	0.63	**0.87**													
10. SysQ	0.60	0.78	0.78	0.74	0.78	0.70	0.64	0.61	0.60	**0.95**												
11. EMP	0.60	0.69	0.70	0.76	0.71	0.57	0.61	0.58	0.57	0.73	**0.92**											
12. SER	0.62	0.68	0.69	0.70	0.74	0.62	0.69	0.61	0.58	0.69	0.74	**0.88**										
13. TAN	0.49	0.71	0.72	0.70	0.74	0.55	0.58	0.59	0.52	0.75	0.66	0.70	**0.93**									
14. ASS	0.54	0.71	0.76	0.75	0.73	0.62	0.57	0.57	0.54	0.75	0.74	0.72	0.78	**0.91**								
15. RES	0.59	0.67	0.69	0.72	0.69	0.61	0.61	0.57	0.61	0.66	0.64	0.72	0.67	0.78	**0**.**94**							
16. SQ	0.57	0.77	0.73	0.68	0.79	0.68	0.65	0.62	0.57	0.75	0.75	0.75	0.72	0.77	0.73	**0.88**						
17. US	0.65	0.79	0.77	0.76	0.79	0.69	0.69	0.68	0.65	0.80	0.78	0.70	0.72	0.76	0.70	0.79	**0.85**					
18. PE	0.41	0.74	0.59	0.53	0.64	0.61	0.51	0.53	0.44	0.65	0.58	0.58	0.57	0.60	0.53	0.70	0.64	**0.94**				
19. PEOU	0.51	0.73	0.62	0.55	0.65	0.69	0.59	0.59	0.51	0.65	0.58	0.58	0.57	0.58	0.54	0.72	0.74	0.68	**0.85**			
20. PU	0.53	0.65	0.62	0.61	0.65	0.79	0.56	0.57	0.49	0.67	0.57	0.55	0.57	0.61	0.54	0.68	0.70	0.58	0.73	**0.93**		
21. BA	0.50	0.75	0.67	0.64	0.73	0.64	0.55	0.59	0.52	0.69	0.61	0.60	0.66	0.64	0.60	0.74	0.78	0.70	0.72	0.73	**0.93**	
22. Intention	0.38	0.64	0.56	0.49	0.53	0.65	0.45	0.42	0.37	0.56	0.51	0.47	0.47	0.53	0.49	0.59	0.62	0.55	0.67	0.69	0.66	**0.95**

The square root of the AVE for each latent variable is displayed in bold. Values below the diagonal line are Pearson’s correlation coefficients.

### 3.3 Structural model

**Fig 2:**
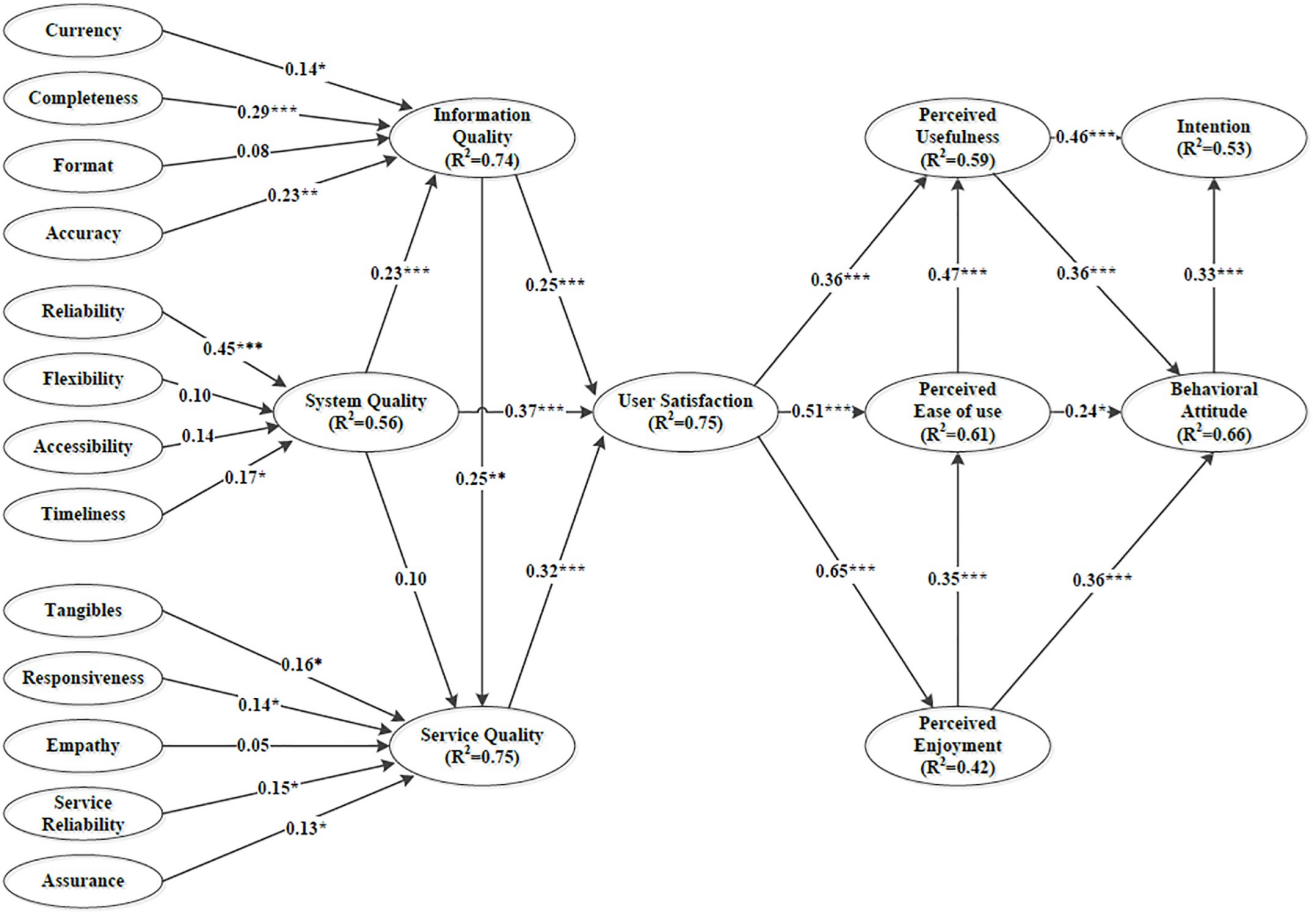
Analysis path of the structural model. Note: *p < 0.05, **p < 0.01, and ***p < 0.001.

The SRMR in this study was 0.056, indicating that the model of this study exhibited a satisfactory model fit. The significant antecedents of IQ were currency (β = 0.14, *p* < 0.05), completeness (β = 0.29, *p* < 0.001), and accuracy (β = 0.23, *p* < 0.01), and those of SysQ were reliability (β = 0.45, *p* < 0.001) and timeliness (β = 0.17, *p* < 0.05). The significant antecedents of SQ were tangibles (β = 0.16, *p* < 0.05), responsiveness (β = 0.14, *p* < 0.05), service reliability (β = 0.15, *p* < 0.05), and assurance (β = 0.13, *p* < 0.05). Fig 2 and Table 4 present the PLS analysis results. PU and BA explained 53% of the variance in intention (R^2^ = 0.53). PU, PEOU, and PE explained 66% of the variance in BA (R^2^ = 0.66). The explained variance (R^2^) of the model was 0.42–0.75, which indicated that the level of the variance explained by the model was higher than moderate.

**Table 4:** Path coefficients, VIF, and results of the research hypotheses.

Hypothesis	Relationship	Path Coefficient	T-Value	Result
H1	SysQ → IQ	0.23***	3.27	Supported
H2	SysQ → SQ	0.10	1.65	Not Supported
H3	IQ → SQ	0.25**	3.35	Supported
H4	SysQ → US	0.37***	5.90	Supported
H5	IQ → US	0.25***	3.42	Supported
H6	SQ → US	0.32***	4.47	Supported
H7	US → PU	0.36***	4.36	Supported
H8	US → PEOU	0.51***	8.45	Supported
H9	US → PE	0.65***	10.23	Supported
H10	PE → PEOU	0.35***	4.95	Supported
H11	PEOU → PU	0.47***	6.16	Supported
H12	PU → Intention	0.46***	5.56	Supported
H13	PU → BA	0.36***	3.60	Supported
H14	PEOU →BA	0.24*	2.48	Supported
H15	PE →BA	0.33***	4.54	Supported
H16	BA → Intention	0.33***	3.69	Supported

*p < 0.05;

**p < 0.01;

***p < 0.001

The PLS analysis results supported 15 of the 16 hypotheses (Fig 2 and Table 4). BA (β = 0.33, *p* < 0.001) and PU (β = 0.46, *p* < 0.001) had significant path coefficients for intention. Hypothesis H2 was not supported (β = 0.10), but according to statistical significance, all other 15 hypotheses were supported.

Table 5 presents the total effect and direct effect of the constructs on intention to use the NPS. PU had the strongest total effect (0.58) and direct effect (0.46) on intention. US had the second strongest total effect (0.53). US had significant direct effects on PE (0.65), PEOU (0.51), and PU (0.36).

**Table 5:** Total and direct effects of the variables on the intention for using the NPS.

Variable	Total effect	Direct effect								
	Intention on intention	IQ	SySQ	SQ	US	PE	PEOU	PU	BA	Intention
CUR	0.02*	0.14*								
COM	0.05**	0.29***								
FOR	0.01	0.08								
ACU	0.04**	0.23***								
IQ	0.17***			0.25**	0.25***					
REL	0.11***		0.45***							
ACE	0.03		0.10							
FLE	0.03		0.14							
TIM	0.04*		0.17*							
SysQ	0.25***	0.23***		0.10	0.37***					
EMP	0.03			0.16*						
SER	0.02			0.14*						
TAN	0.01			0.05						
ASS	0.02			0.15*						
RES	0.03			0.13*						
SQ	0.17***				0.32***					
US	0.53***					0.65***	0.51***	0.36***		
PE	0.23***						0.35***		0.33***	
PEOU	0.35***							0.47***	0.24*	
PU	0.58***								0.36***	0.46***
BA	0.33***									0.33***

*p < 0.05;

**p < 0.01;

***p < 0.001

## 4 Discussion

### 4.1 Principal findings

The results supported 15 of our hypotheses, validating the 3Q model proposed by Xu, Benbasat [10]. This model can be employed to understand the behavioral intention of nurses toward using the NPS. We successfully used technology acceptance and US to explore the factors affecting intention to use the NPS. When designing an NIS, the behavioral beliefs of nurses regarding technology acceptance must be considered to ensure they will use the system. Moreover, the results indicated that SQ, IQ, and SysQ affected US, which had strong total effects on intention. This study is the first to indicate that the 3Q model crucially indicates whether nurses use an NPS.

### 4.2 Comparison with prior work

Regarding behavioral beliefs, PU had the strongest total effect (0.58) and direct effect (0.46) on intention. This strongest total effect of PU on intention was influenced by the paths from PEOU to PU and from US to PU. The results indicated that the system may assist nurses in providing clinical care; thus, nurses strongly perceive the system’s usefulness. Furthermore, PU was affected by PEOU, which is consistent with the results of previous studies [8, 10, 28–33]. The total effect of PU on intention (0.58) was stronger than that of PEOU (0.35), which is consistent with reported findings [8, 10, 29–32, 34, 35]. When users quickly find the function they require, they perceive the information system as being easy to use, which increases the system’s usefulness. However, some studies have demonstrated that PEOU has a stronger influence on intention than does PU [28, 33, 36, 37]. This difference between the results of our study and those of the aforementioned studies may be because the NPS investigated in this study provides access to medical information and assists nurses in establishing and recording the daily care plan of patients, helping them perform their care-related tasks. In summary, when nurses have a positive perception of the usefulness and ease of use of the NIS, they can perform their work with high efficiency and have a high intention to use the system.

US exerted the second strongest total effect on intention, which indicated that US affected intention through the behavioral belief of technology acceptance. Our research results indicated that when users found the information system enjoyable and easy to use, they had a positive BA. Thus, US influenced the degree of acceptance of the information system. Users were more satisfied with the performance of the information system when they had a stronger perception of the system being useful and easy to use [8]. The present study focused on the NPS and did not analyze the information success of the clinical decision support system. We recommend exploring the user perceptions of nursing process decision support systems through object-based beliefs (SysQ, IQ, and SQ) in the future.

In this study, SysQ, IQ, and SQ explained 75% of the variance in US (R^2^ = 0.75). SysQ, IQ, and SQ are critical constructs for exploring the intention of NPS users. The NIS should be designed considering information-related quality. For the NIS, IQ, SQ, and SysQ can only be obtained from nurses. In clinical institutions, programmers and users often identify inconsistencies in a system’s function and requirements. Lu, Hsiao [34] investigated nurses’ acceptance of a hospital information system and discovered that SysQ, IQ, and SQ positively influenced PEOU and PU. According to previous studies, the overall support provided by an information system department can be assessed by examining SQ [10, 38], which has been widely applied in offline and new online domains [10]. Using types of information-related quality, especially SQ, to evaluate medical information systems has not been widely discussed. We established 16 hypotheses on the basis of the 3Q model and analyzed the influence of SysQ, IQ, and SQ on US.

Medical information systems are not yet completely paperless. Thus, nursing personnel must enter patient information and then print medical records by using printers or other equipment. When nurses experience a setback, their evaluation of the quality of the information system is affected. Senior staff can use the system easily because of their clinical experience and ability. When using the NPS to establish a care plan, nurses rely on their personal knowledge, care skills, and experience. New staff members require the assistance of others or relevant information because of their lack of experience. Therefore, the impact of SysQ on SQ may be nonsignificant.

## 5 Limitations

Our study used the extended WT model to construct a 3Q model, which was originally proposed by Xu, Benbasat [10]; only one model was discussed and used as our research framework, which may have decreased the strength of this research. Furthermore, we referred to the information system success model employed in medical information system research [19] and combined service satisfaction, information satisfaction, and system satisfaction to determine the effect of US on intention to use the NPS. Because this is inconsistent with the research by Xu et al.Xu, Benbasat [10], we could not determine the influence of object-based beliefs on each object-based attitude. Future research should consider these relationships when constructing the 3Q model.

The second limitation of this study is that it had a cross-sectional design, and the data were collected at only one time point. Longitudinal studies conducted in other information system fields have examined the moderating effects on the relationships between latent constructs [39, 40]. We recommend that in the future, scholars should explore the effects of the moderators, such as user experience and voluntary use.

Another limitation of this study is that we proposed the constructs of IQ and SysQ before those of SQ to explore user behavior intention toward the NPS. The results did not show the effects among the constructs of object-based beliefs and incompletely represent nurses’ perceptions of SQ and its effect on SysQ. Future research should consider the influence of the services provided by the programmer on SysQ and IQ and the changes that may occur in the service environment.

## 6 Conclusion

We investigated the intention of nursing staff to use the NPS by employing a 3Q (IQ, SQ, and SysQ) model that integrated US and technology acceptance. Our study provides a methodology for exploring users’ beliefs, attitudes, and intentions.

In this study, we obtained empirical evidence to determine the critical factors influencing nurses’ perceptions of the NPS. To increase the intention of users, NPS designers must consider the workflow of care-related tasks, features of nursing routines, and nurses’ requirements to design and implement an NIS that satisfies users and is easy to use. Furthermore, programmers and designers must attach considerable importance to IQ, SQ, and SysQ to develop a successful NIS. To control the characteristics of the NPS and satisfy users, the system should be designed to be appropriate and user friendly and to assist nurses in their duties.

## Supporting information

S1 File Questionnaire in Chinese. (DOCX)

S2 File Questionnaire in English. (DOCX)

S3 File Relevant data. (CSV)
