# Supplementary material for: Theoretical integration of user satisfaction and technology acceptance of the nursing process information system
Source: PLoS One. 2019 Jun 4;14(6):e0217622. doi: 10.1371/journal.pone.0217622 (PMC6548361; doi:10.1371/journal.pone.0217622)
Supplement: S1 File — (DOCX) [file pone.0217622.s001.docx]

一、基本資料

1. 性別：□男□女
2. 教育程度：□高職□副學士□學士□碩士
3. 護理職稱：□護理師□護理長
4. 護理工作年資：□0-5年□6-10年□11-15年□16-20年□20年以上
5. 醫療資訊系統使用年資：□0-5年□6-10年□10年以上
6. 使用資訊系統是否感覺有壓力： □是□否

二、請您就使用護理過程資訊系統之經驗，填寫下列問卷。

| 項目 | | 強烈  不同意 | 沒意見 | 強烈  同意 | |
| --- | --- | --- | --- | --- | --- |
| 此系統提供我工作上最近的訊息 | | **-5 -4 -3 -2 -1 0 1 2 3 4 5** | | | |
| 從此系統可展現工作上最新訊息 | | **-5 -4 -3 -2 -1 0 1 2 3 4 5** | | | |
| 此系統的訊息總是保持即時更新的狀態 | | **-5 -4 -3 -2 -1 0 1 2 3 4 5** | | | |
| 此系統提供我完整的工作所需訊息 | | **-5 -4 -3 -2 -1 0 1 2 3 4 5** | | | |
| 此系統產生與我工作相關的廣泛訊息 | | **-5 -4 -3 -2 -1 0 1 2 3 4 5** | | | |
| 此系統提供我在工作上所有需要的訊息 | | **-5 -4 -3 -2 -1 0 1 2 3 4 5** | | | |
| 此系統提供的訊息符合工作所需的格式 | | **-5 -4 -3 -2 -1 0 1 2 3 4 5** | | | |
| 此系統提供的訊息格式是依據工作內容設計 | | **-5 -4 -3 -2 -1 0 1 2 3 4 5** | | | |
| 此系統介面呈現清楚工作上訊息 | | **-5 -4 -3 -2 -1 0 1 2 3 4 5** | | | |
| 此系統可提供正確訊息供工作使用 | | **-5 -4 -3 -2 -1 0 1 2 3 4 5** | | | |
| 我從此系統所得到工作相關的訊息是零錯誤 | | **-5 -4 -3 -2 -1 0 1 2 3 4 5** | | | |
| 此系統精確的提供工作相關訊息 | | **-5 -4 -3 -2 -1 0 1 2 3 4 5** | | | |
| 針對此系統提供工作上的訊息，我給予高度評價 | | **-5 -4 -3 -2 -1 0 1 2 3 4 5** | | | |
| 整體而言，對於此系統上提供關於工作所需的訊息，我給予高分 | | **-5 -4 -3 -2 -1 0 1 2 3 4 5** | | | |
| 基本上，此系統提供我高品質的工作相關訊息 | | **-5 -4 -3 -2 -1 0 1 2 3 4 5** | | | |
| 整體而言，對於從此系統獲得關於工作所需的訊息，我很滿意 | | **-5 -4 -3 -2 -1 0 1 2 3 4 5** | | | |
| 我很滿意此系統上所呈現的訊息 | | **-5 -4 -3 -2 -1 0 1 2 3 4 5** | | | |
| 對協助工作而言，此系統提供了非常令人滿意的訊息 | | **-5 -4 -3 -2 -1 0 1 2 3 4 5** | | | |
| 使用此系統來進行我的工作時，我認為系統運作是可靠的 | | **-5 -4 -3 -2 -1 0 1 2 3 4 5** | | | |
| 在我進行工作時，此系統可穩定運作 | | **-5 -4 -3 -2 -1 0 1 2 3 4 5** | | | |
| 我可以依賴此系統的運作來進行工作。 | | **-5 -4 -3 -2 -1 0 1 2 3 4 5** | | | |
| 在工作時，此系統我可以立即取得 | | **-5 -4 -3 -2 -1 0 1 2 3 4 5** | | | |
| 在工作時，此系統是容易就近使用的 | | **-5 -4 -3 -2 -1 0 1 2 3 4 5** | | | |
| 在工作時，很容易連結進此系統 | | **-5 -4 -3 -2 -1 0 1 2 3 4 5** | | | |
| 此系統可彈性應用，以滿足我在工作上的各種需求 | | **-5 -4 -3 -2 -1 0 1 2 3 4 5** | | | |
| 此系統能夠靈活地調整成我在工作上的要求或情況 | | **-5 -4 -3 -2 -1 0 1 2 3 4 5** | | | |
| 此系統十分靈活的滿足我在工作上的需要 | | **-5 -4 -3 -2 -1 0 1 2 3 4 5** | | | |
| 此系統花了很多時間來回應我在工作上的需求 | | **-5 -4 -3 -2 -1 0 1 2 3 4 5** | | | |
| 此系統能及時回應我在工作上的需求 | | **-5 -4 -3 -2 -1 0 1 2 3 4 5** | | | |
| 進行工作時，此系統能很快回應我的要求 | | **-5 -4 -3 -2 -1 0 1 2 3 4 5** | | | |
| 在系統質量方面，我給予此系統高評價 | | **-5 -4 -3 -2 -1 0 1 2 3 4 5** | | | |
| 就我的工作而言，我所使用的這個系統是具有良好品質 | | **-5 -4 -3 -2 -1 0 1 2 3 4 5** | | | |
| 就我的工作而言，我會給此系統的高品質的評價 | | **-5 -4 -3 -2 -1 0 1 2 3 4 5** | | | |
| 經過周全的思考後，我對此系統感到非常滿意 | | **-5 -4 -3 -2 -1 0 1 2 3 4 5** | | | |
| 整體而言，在工作時與此系統的互動我很滿意 | | **-5 -4 -3 -2 -1 0 1 2 3 4 5** | | | |
| 我很滿意使用此系統進行我的工作 | | **-5 -4 -3 -2 -1 0 1 2 3 4 5** | | | |
| 進行工作時，此系統能提供我在工作上個別性需求 | | **-5 -4 -3 -2 -1 0 1 2 3 4 5** | | | |
| 我對此系統有很大的興趣 | | **-5 -4 -3 -2 -1 0 1 2 3 4 5** | | | |
| 此系統的運用機制符合我工作上的個別化的操作 | | **-5 -4 -3 -2 -1 0 1 2 3 4 5** | | | |
| 此系統瞭解我在工作上的特定需求 | | **-5 -4 -3 -2 -1 0 1 2 3 4 5** | | | |
| 我相信在工作時，此系統會依我要求提供服務 | | **-5 -4 -3 -2 -1 0 1 2 3 4 5** | | | |
| 在工作時，此系統能提供正確的服務 | | **-5 -4 -3 -2 -1 0 1 2 3 4 5** | | | |
| 在工作時，此系統可以提供即時回應服務 | | **-5 -4 -3 -2 -1 0 1 2 3 4 5** | | | |
| 此系統是更新為最新的。 | | **-5 -4 -3 -2 -1 0 1 2 3 4 5** | | | |
| 此系統介面具有視覺上的吸引力。 | | **-5 -4 -3 -2 -1 0 1 2 3 4 5** | | | |
| 此系統介面整潔美觀。 | | **-5 -4 -3 -2 -1 0 1 2 3 4 5** | | | |
| 此系統介面符合它所提供的服務。 | | **-5 -4 -3 -2 -1 0 1 2 3 4 5** | | | |
| 我有信心此系統協助我在工作上做出的決策。 | | **-5 -4 -3 -2 -1 0 1 2 3 4 5** | | | |
| 有此系統協助工作，讓我感到安心。 | | **-5 -4 -3 -2 -1 0 1 2 3 4 5** | | | |
| 此系統能回應我工作上的所有問題。 | | **-5 -4 -3 -2 -1 0 1 2 3 4 5** | | | |
| 我相信，此系統能回應我工作上的需求。 | | **-5 -4 -3 -2 -1 0 1 2 3 4 5** | | | |
| 在任何問題之下，我認為此系統可迅速提供協助。 | | **-5 -4 -3 -2 -1 0 1 2 3 4 5** | | | |
| 此系統回應了我在工作中所有任何顧慮 | | **-5 -4 -3 -2 -1 0 1 2 3 4 5** | | | |
| 從此系統我得到「好」的服務品質 | | **-5 -4 -3 -2 -1 0 1 2 3 4 5** | | | |
| 從此系統我得到「非常優良」的服務品質 | | **-5 -4 -3 -2 -1 0 1 2 3 4 5** | | | |
| 從此系統我得到「高品質」的服務 | | **-5 -4 -3 -2 -1 0 1 2 3 4 5** | | | |
| 我對從此系統獲得的服務感到非常滿意 | | **-5 -4 -3 -2 -1 0 1 2 3 4 5** | | | |
| 我很滿意，進行工作時從此系統上獲得的服務 | | **-5 -4 -3 -2 -1 0 1 2 3 4 5** | | | |
| 就進行工作而言，此系統提供的服務是非常令人滿意的 | | **-5 -4 -3 -2 -1 0 1 2 3 4 5** | | | |
| 使用此系統進行工作是愉快的 | | **-5 -4 -3 -2 -1 0 1 2 3 4 5** | | | |
| 使用此系統進行工作是令人興奮的 | | **-5 -4 -3 -2 -1 0 1 2 3 4 5** | | | |
| 使用此系統進行工作是很有趣的 | | **-5 -4 -3 -2 -1 0 1 2 3 4 5** | | | |
| 使用此系統進行工作是開心 | | **-5 -4 -3 -2 -1 0 1 2 3 4 5** | | | |
| 使用此系統進行工作是愉悅的 | | **-5 -4 -3 -2 -1 0 1 2 3 4 5** | | | |
| 操作此系統做我想做的事情是容易的 | | **-5 -4 -3 -2 -1 0 1 2 3 4 5** | | | |
| 我發現使用此系統來進行我的工作是容易的 | | **-5 -4 -3 -2 -1 0 1 2 3 4 5** | | | |
| 對我來說使用此系統進行工作是作容易的事 | | **-5 -4 -3 -2 -1 0 1 2 3 4 5** | | | |
| 學習操作此系統是件容易的事 | | **-5 -4 -3 -2 -1 0 1 2 3 4 5** | | | |
| 我可以明確且了解如何去操作此系統 | | **-5 -4 -3 -2 -1 0 1 2 3 4 5** | | | |
| 使用此系統可以增加我的工作生產力 | | **-5 -4 -3 -2 -1 0 1 2 3 4 5** | | | |
| 使用此系統有用於我的工作進行 | | **-5 -4 -3 -2 -1 0 1 2 3 4 5** | | | |
| 使用此系統提高了我的工作效率 | | **-5 -4 -3 -2 -1 0 1 2 3 4 5** | | | |
| 使用此系統可以提升我的工作表現。 | | **-5 -4 -3 -2 -1 0 1 2 3 4 5** | | | |
| 經過周全的思考後，使用此系統進行工作是不錯的主意 | | **-5 -4 -3 -2 -1 0 1 2 3 4 5** | | | |
| 經過周全的思考後，使用此系統進行工作是明智之舉 | | **-5 -4 -3 -2 -1 0 1 2 3 4 5** | | | |
| 經過周全的思考後，使用此系統進行工作是積極的步驟 | | **-5 -4 -3 -2 -1 0 1 2 3 4 5** | | | |
| 經過周全的思考後，使用此系統進行工作是有效率的想法 | | **-5 -4 -3 -2 -1 0 1 2 3 4 5** | | | |
| 下一次工作時，我希望能使用此系統 | | **-5 -4 -3 -2 -1 0 1 2 3 4 5** | | | |
| 如果可以使用此系統，未來我有意願使用它來進行工作 | | **-5 -4 -3 -2 -1 0 1 2 3 4 5** | | | |
| 如果可以使用此系統，未來我預計會想要使用它來進行工作 | | **-5 -4 -3 -2 -1 0 1 2 3 4 5** | | | |
