# Supplementary material for: Theoretical integration of user satisfaction and technology acceptance of the nursing process information system
Source: PLoS One. 2019 Jun 4;14(6):e0217622. doi: 10.1371/journal.pone.0217622 (PMC6548361; doi:10.1371/journal.pone.0217622)
Supplement: S2 File — (DOCX) [file pone.0217622.s002.docx]

Part I : Personal Data

1. Gender : □ Male □Female
2. Education level : □Senior vocational school □Associate degree □Bachelor’s degree □Master’s degree
3. Position: □Staff □Supervisor
4. Experience of nursing staff(y) : □0–5 □6–10 □ 11–15 □16–20 □>20
5. Experience of using Hospital Information System (y): □0-5 □6-10 □>10
6. Experiencing pressure when using a computer: □Yes □No

Part II : Please fill out every question in the following questionnaire according to your experiences of using nursing process system.

| Item | | completely disagree |  | completely agree | |
| --- | --- | --- | --- | --- | --- |
| The system provided me with the most recent information for my task. | | **-5 -4 -3 -2 -1 0 1 2 3 4 5** | | | |
| The system produced the most current information for my task. | | **-5 -4 -3 -2 -1 0 1 2 3 4 5** | | | |
| The information from the system was always up to date for my task. | | **-5 -4 -3 -2 -1 0 1 2 3 4 5** | | | |
| The system provided me with a complete set of information for my task. | | **-5 -4 -3 -2 -1 0 1 2 3 4 5** | | | |
| The system produced comprehensive information for my task. | | **-5 -4 -3 -2 -1 0 1 2 3 4 5** | | | |
| The system provided me with all the information I needed for my task. | | **-5 -4 -3 -2 -1 0 1 2 3 4 5** | | | |
| The information provided by the system was well formatted for my task. | | **-5 -4 -3 -2 -1 0 1 2 3 4 5** | | | |
| The information provided by the system was well laid out for my task. | | **-5 -4 -3 -2 -1 0 1 2 3 4 5** | | | |
| The information provided by the system was clearly presented on the screen for my task. | | **-5 -4 -3 -2 -1 0 1 2 3 4 5** | | | |
| The system produced correct information for my task. | | **-5 -4 -3 -2 -1 0 1 2 3 4 5** | | | |
| The information I obtained from the system for my task was error-free. | | **-5 -4 -3 -2 -1 0 1 2 3 4 5** | | | |
| The information provided by the system was accurate for my task. | | **-5 -4 -3 -2 -1 0 1 2 3 4 5** | | | |
| Overall, I would give the information from the system high marks for my task. | | **-5 -4 -3 -2 -1 0 1 2 3 4 5** | | | |
| Overall, I would give the information provided by the system a high rating in terms of quality for my task. | | **-5 -4 -3 -2 -1 0 1 2 3 4 5** | | | |
| In general, the system provided me with high-quality information for my task. | | **-5 -4 -3 -2 -1 0 1 2 3 4 5** | | | |
| Overall, the information I got from the system was very satisfying to execute my task. | | **-5 -4 -3 -2 -1 0 1 2 3 4 5** | | | |
| I am very satisfied with the information I received from the system to execute my task.. | | **-5 -4 -3 -2 -1 0 1 2 3 4 5** | | | |
| The system provided very satisfactory information for me to execute my task. | | **-5 -4 -3 -2 -1 0 1 2 3 4 5** | | | |
| The system operated reliably for my task. | | **-5 -4 -3 -2 -1 0 1 2 3 4 5** | | | |
| The system performed reliably for my task. | | **-5 -4 -3 -2 -1 0 1 2 3 4 5** | | | |
| The operation of the system was dependable for my task. | | **-5 -4 -3 -2 -1 0 1 2 3 4 5** | | | |
| The system was readily accessible to me in my task. | | **-5 -4 -3 -2 -1 0 1 2 3 4 5** | | | |
| The system was very accessible during my task. | | **-5 -4 -3 -2 -1 0 1 2 3 4 5** | | | |
| The system was easy to access during my task. | | **-5 -4 -3 -2 -1 0 1 2 3 4 5** | | | |
| The system was able to be adapted to meet a variety of needs during my task. | | **-5 -4 -3 -2 -1 0 1 2 3 4 5** | | | |
| The system was able to flexibly adjust to new demands or conditions during my task. | | **-5 -4 -3 -2 -1 0 1 2 3 4 5** | | | |
| The system was flexible in addressing needs as they arise during my task. | | **-5 -4 -3 -2 -1 0 1 2 3 4 5** | | | |
| It took too long for the system to respond to my requests during my task. | | **-5 -4 -3 -2 -1 0 1 2 3 4 5** | | | |
| The system responded in a timely fashion during my task. | | **-5 -4 -3 -2 -1 0 1 2 3 4 5** | | | |
| The system answered my requests quickly during my task. | | **-5 -4 -3 -2 -1 0 1 2 3 4 5** | | | |
| In terms of system quality, I would rate the system highly for my task. | | **-5 -4 -3 -2 -1 0 1 2 3 4 5** | | | |
| Overall, the system that I used was of high quality for my task. | | **-5 -4 -3 -2 -1 0 1 2 3 4 5** | | | |
| Overall, I would give the quality of the system a high rating for my task. | | **-5 -4 -3 -2 -1 0 1 2 3 4 5** | | | |
| All things considered, I am very satisfied with the system to execute my task. | | **-5 -4 -3 -2 -1 0 1 2 3 4 5** | | | |
| Overall, my interaction with the system to execute my task was very satisfying. | | **-5 -4 -3 -2 -1 0 1 2 3 4 5** | | | |
| The system was very satisfying for me to execute my task. | | **-5 -4 -3 -2 -1 0 1 2 3 4 5** | | | |
| The system gave me individual attention during my task. | | **-5 -4 -3 -2 -1 0 1 2 3 4 5** | | | |
| The system had my best interests in mind during my task. | | **-5 -4 -3 -2 -1 0 1 2 3 4 5** | | | |
| The system had mechanisms that gave me personal attention during my task. | | **-5 -4 -3 -2 -1 0 1 2 3 4 5** | | | |
| The system understood my specific needs during my task. | | **-5 -4 -3 -2 -1 0 1 2 3 4 5** | | | |
| I believe that what I asked for was what I got during my task in the system. | | **-5 -4 -3 -2 -1 0 1 2 3 4 5** | | | |
| The system performed the service right during my task. | | **-5 -4 -3 -2 -1 0 1 2 3 4 5** | | | |
| The system provided its service at the time it promised to do so during my task. | | **-5 -4 -3 -2 -1 0 1 2 3 4 5** | | | |
| The system was up to date. | | **-5 -4 -3 -2 -1 0 1 2 3 4 5** | | | |
| The system was visually appealing. | | **-5 -4 -3 -2 -1 0 1 2 3 4 5** | | | |
| The system was neat in appearance. | | **-5 -4 -3 -2 -1 0 1 2 3 4 5** | | | |
| The appearance of the system was in keeping with the services it provides. | | **-5 -4 -3 -2 -1 0 1 2 3 4 5** | | | |
| I felt confident about the decision for my task in the system. | | **-5 -4 -3 -2 -1 0 1 2 3 4 5** | | | |
| I felt safe in my interaction with the system during my task. | | **-5 -4 -3 -2 -1 0 1 2 3 4 5** | | | |
| The system had answers to all my questions about my task. | | **-5 -4 -3 -2 -1 0 1 2 3 4 5** | | | |
| I believe the system was responsive to my needs during my task. | | **-5 -4 -3 -2 -1 0 1 2 3 4 5** | | | |
| In the case of any problem, I think the system would give me prompt service during my task. | | **-5 -4 -3 -2 -1 0 1 2 3 4 5** | | | |
| The system addressed any concerns that I had during my task. | | **-5 -4 -3 -2 -1 0 1 2 3 4 5** | | | |
| Overall, the level of service quality I received from the system during my task was good. | | **-5 -4 -3 -2 -1 0 1 2 3 4 5** | | | |
| Overall, the level of service quality I received from the system during my task was excellent. | | **-5 -4 -3 -2 -1 0 1 2 3 4 5** | | | |
| Overall, the level of service quality I received from the system during my task was high. | | **-5 -4 -3 -2 -1 0 1 2 3 4 5** | | | |
| Overall, the service I received from the system was very satisfying to execute my task. | | **-5 -4 -3 -2 -1 0 1 2 3 4 5** | | | |
| I am very satisfied with the service I received from the system to execute my task. | | **-5 -4 -3 -2 -1 0 1 2 3 4 5** | | | |
| In terms of my task, the service provided by the system was very satisfying. | | **-5 -4 -3 -2 -1 0 1 2 3 4 5** | | | |
| Using the system to execute my task was enjoyable. | | **-5 -4 -3 -2 -1 0 1 2 3 4 5** | | | |
| Using the system to execute my task was exciting. | | **-5 -4 -3 -2 -1 0 1 2 3 4 5** | | | |
| Using the system to execute my task was interesting. | | **-5 -4 -3 -2 -1 0 1 2 3 4 5** | | | |
| Using the system to execute my task was fun. | | **-5 -4 -3 -2 -1 0 1 2 3 4 5** | | | |
| Using the system to execute my task was pleasant. | | **-5 -4 -3 -2 -1 0 1 2 3 4 5** | | | |
| It was easy to get the system to do what I wanted it to do. | | **-5 -4 -3 -2 -1 0 1 2 3 4 5** | | | |
| Overall, I found that the system was easy to use to execute my task. | | **-5 -4 -3 -2 -1 0 1 2 3 4 5** | | | |
| It was easy for me to execute my task using the system. | | **-5 -4 -3 -2 -1 0 1 2 3 4 5** | | | |
| Learning to use the system to execute my task was easy. | | **-5 -4 -3 -2 -1 0 1 2 3 4 5** | | | |
| My interaction with the system to execute my task was clear and understandable. | | **-5 -4 -3 -2 -1 0 1 2 3 4 5** | | | |
| Using the system to execute my task increased my productivity in my task. | | **-5 -4 -3 -2 -1 0 1 2 3 4 5** | | | |
| I found the system useful in my task. | | **-5 -4 -3 -2 -1 0 1 2 3 4 5** | | | |
| Using the system enhanced the effectiveness in my task. | | **-5 -4 -3 -2 -1 0 1 2 3 4 5** | | | |
| Using the system improved the performance in my task. | | **-5 -4 -3 -2 -1 0 1 2 3 4 5** | | | |
| All things considered, using the system to execute my task will be a good idea. | | **-5 -4 -3 -2 -1 0 1 2 3 4 5** | | | |
| All things considered, using the system to execute my task will be a wise move. | | **-5 -4 -3 -2 -1 0 1 2 3 4 5** | | | |
| All things considered, using the system to execute my task will be a positive step. | | **-5 -4 -3 -2 -1 0 1 2 3 4 5** | | | |
| All things considered, using the system to execute my task will be an effective idea. | | **-5 -4 -3 -2 -1 0 1 2 3 4 5** | | | |
| Next time I need to execute my task, I would like to use this system. | | **-5 -4 -3 -2 -1 0 1 2 3 4 5** | | | |
| Assuming I had access to the system, I intend to use it to execute my task in the future. | | **-5 -4 -3 -2 -1 0 1 2 3 4 5** | | | |
| Given that I had access to the system, I predict that I would use it to execute my task in the future. | | **-5 -4 -3 -2 -1 0 1 2 3 4 5** | | | |
